# A Preliminary Survey of Transfer RNA Modifications and Modifying Enzymes of the Tropical Plant *Cocos nucifera* L.

**DOI:** 10.3390/genes14061287

**Published:** 2023-06-18

**Authors:** Meng Chu, Yichao Qin, Xiuying Lin, Li Ma, Dehai Deng, Daizhu Lv, Pengcheng Fu, Huan Lin

**Affiliations:** 1 State Key Laboratory of Marine Resource Utilization in South China Sea, Hainan University, Haikou 570228, China; 2 School of Life Sciences, Hainan University, Haikou 570228, China; 3 College of Ecology and Environment, Hainan University, Haikou 570228, China; 4 Analysis and Testing Center, Chinese Academy of Tropical Agricultural Sciences, Haikou 571101, China

**Keywords:** *Cocos nucifera*, epitranscriptomics, LC-MS/MS, nucleoside misidentification, RNA modification

## Abstract

The coconut (*Cocos nucifera* L.) is a commercial crop widely distributed among coastal tropical regions. It provides millions of farmers with food, fuel, cosmetics, folk medicine, and building materials. Among these, oil and palm sugar are representative extracts. However, this unique living species of *Cocos* has only been preliminarily studied at molecular levels. Benefiting from the genomic sequence data published in 2017 and 2021, we investigated the transfer RNA (tRNA) modifications and modifying enzymes of the coconut in this survey. An extraction method for the tRNA pool from coconut flesh was built. In total, 33 species of modified nucleosides and 66 homologous genes of modifying enzymes were confirmed using a nucleoside analysis using high-performance liquid chromatography combined with high-resolution mass spectrometry (HPLC-HRMS) and homologous protein sequence alignment. The positions of tRNA modifications, including pseudouridines, were preliminarily mapped using a oligonucleotide analysis, and the features of their modifying enzymes were summarized. Interestingly, we found that the gene encoding the modifying enzyme of 2′-O-ribosyladenosine at the 64th position of tRNA (Ar(p)64) was uniquely overexpressed under high-salinity stress. In contrast, most other tRNA-modifying enzymes were downregulated with mining transcriptomic sequencing data. According to previous physiological studies of Ar(p)64, the coconut appears to enhance the quality control of the translation process when subjected to high-salinity stress. We hope this survey can help advance research on tRNA modification and scientific studies of the coconut, as well as thinking of the safety and nutritional value of naturally modified nucleosides.

## 1. Introduction

Nucleoside analogs are artificially modified or synthesized based on the natural nucleosides adenosine, uridine, cytidine, and guanosine. As they mimic the RNA building blocks and could be incorporated into nascent messenger RNA (mRNA), some are used as transcriptional inhibitors to treat cancer and virus infection, including the COVID-19 drugs Molnupiravir [1] and Remdesivir [2]. On the other hand, naturally modified nucleosides inherently exist. They are modified post-transcriptionally on small or large RNA elements, including mRNA, transfer RNA (tRNA), micro-RNA (miRNA), and ribosomal RNA (rRNA), among others. After spontaneously degrading from RNA elements, they become free-modified nucleosides in a cell. Over 160 species of post-transcriptional RNA modifications have been found in all three kingdoms of life to date, 70% of which have been identified on tRNAs [3].

An increasing focus on the post-transcriptional modification of RNA and its critical impact on disease development, polypeptide translation quality control, and efficiency has been given to RNA subcellular localization, RNA–protein interactions, and RNA degradation research [4,5,6]. It has been shown in recent investigations of epitranscriptomes that dynamic RNA modification is a response to physiological and environmental changes [6,7,8]. For instance, modifications at positions 34 (the wobble position), 37, and 58 of tRNAs can be related to environmental factors. cmo^5^U34 of tRNA^Thr(UGU)^ (uridine 5-oxyacetic acid on position 34) is enhanced with dissolved oxygen and advances the reading of ACG codons [9]. t^6^A37 (*N*^6^-threonylcarbamoyladenosine) of mitochondrial tRNAs decoding ANN codons responds to the bicarbonate concentration, whereas m^1^A58 (1-methyladenosine) of cytoplasmic tRNAs responds to the glucose concentration of the culture medium via an FTO (m^1^A eraser)-dependent pathway, respectively, in human cell lines [10,11]. The dynamic m^6^A (*N*^6^-methyladenosine) modification of mRNAs has been intensively investigated. YTHDF2 (an m^6^A reader) and FTO (an m^6^A eraser) competitively bind to the 5’-UTR of mRNA and modify m^6^A methylation in a mouse embryonic fibroblast (MEF), enabling cap-unbiased translation of particular transcripts beneath stress conditions [12]. m^6^A has also been vital in initiating meiosis at some stages of nitrogen starvation in budding yeast [13]. Flexible ho^5^C2501 (5-hydroxycytidine) on 23S rRNA helps *Escherichia coli* protect against oxidative stress [14].

The roles of RNA modifications in specific positions are being uncovered. However, the toxicity and physiological functions of free-modified nucleosides degraded from RNA elements are poorly understood. A few were found to work as signal molecules. The i^6^A (*N*^6^-isopentenyladenosine, *N*^6^-isopentenyladenine riboside, or iPR), io^6^A (*N*^6^-cis-hydroxyisopentenyladenosine, cis-Zeatin riboside, or cZR), ms^2^i^6^A (2-methylthio-*N*^6^-isopentenyladenosine, 2-methylthio-*N*^6^-isopentenyladenine riboside, or 2MeSiPR), ms^2^io^6^A (2-methylthio-*N*^6^-cis-hydroxyisopentenyladenosine, 2-methylthio-cis-Zeatin riboside, or 2MeScZR) degrade from tRNAs, and their derivatives iP (*N*^6^-isopentenyladenine), cZ (cis-Zeatin), and 2MeScZ (2-methylthio-cis-Zeatin) are members of plant hormone cytokinins that promote cell division in plant roots and shoots [15,16]. Free m^6^A was found to be an endogenous adenosine A3 receptor ligand that facilitates allergy and inflammation in human cell lines [17]. Free i^6^A was also toxic to human cancer cell lines. Extracellular i^6^A addition to a cell culture medium results in i^6^A incorporation into rRNAs in the 5-fluorouracil (5-FU)-resistant human oral squamous cell carcinoma cell line FR2-SAS and its parental 5-FU-sensitive cell line SAS [18]. These free-modified nucleosides may be absorbed by the daily animal and vegetal diets and microbial food sources. Nonetheless, their biological roles are unclear, and more information on free-modified nucleosides should be researched. Surveying RNA modifications of dietary food species is essential, as it can help improve RNA modification studies and food safety and nutrition science.

Some studies of RNA modification mapping to model plants, such as Arabidopsis thaliana, have been published [19,20,21,22]. Understanding the biological roles of this epitranscriptomic information in plants could help us uncover deeper translation regulation mechanisms in response to environmental change. We chose the coconut (*C. nucifera* L.) as a template for this survey of tRNA modifications and modifying enzymes. The coconut is a traditional tropical food and a unique living species of the monospecific genus *Cocos*. It is widely cultivated in coastal tropical regions. Copra, oil, palm sugar, fibrous shells, cosmetics, folk medicine, and building materials are processed and extracted from this valuable plant. However, because of its long cultivation period (the “Tall” type and “Dwarf” type flower in 8–10 years or 4–6 years after planting, respectively), progress in breeding efforts and molecular biology research has been extremely slow. It is reassuring to see that coconut genome sequencing data were published in 2017 and 2021 [23,24]. Following these previous works, we established an effective method to isolate pure tRNA pools (rich in modified nucleosides) from coconut flesh. Then, 33 species of modified nucleosides and 66 homologous genes of modifying enzymes were identified with a nucleoside analysis using high-performance liquid chromatography combined with high-resolution mass spectrometry (HPLC-HRMS) and homologous protein sequence alignment. Two hundred and forty-one tRNA genes (supplementary materials) were found by searching in the genome of the coconut using an online server called tRNAscan-SE 2.0 with manual checking [25]. The positions of tRNA modifications, including pseudouridines, were preliminarily mapped with an oligonucleotide analysis and RNA ModMapper [26]. Features of their modifying enzymes were summarized. 

Interestingly, by mining transcriptome data published previously [24], we found that the gene encoding the modifying enzyme of 2′-O-ribosyladenosine on the 64th position of tRNA (Ar(p)64) was uniquely overexpressed under high-salinity stress, whereas most other tRNA-modifying enzymes were downregulated. According to previous physiological studies of Ar(p)64 [27], the coconut appears to enhance quality control of the translation process when subjected to high-salinity stress. 

## 2. Materials and Methods

### 2.1. Materials and Reagents

Mature coconuts (Hainan Tall, ripening 11–13 months) were purchased from a local market in Haikou (Hainan Island, China). Mature coconuts have a dry brown shell with little moisture in the coir cavity, a thick edible endosperm (flesh or meat) of about 13 mm, and a thin brown seed coat (testa). 

Nucleoside standards were bought from reputable business producers or agencies. Adenosine (A), uridine (U), cytidine (C), guanosine (G), inosine (I), *N*^6^-methyladenosine (m^6^A), 2′-O-methyluridine (Um), 5-methyluridine (m^5^U), 2′-O-methylcytidine (Cm), and 2′-O-methylguanosine (Gm) were obtained from Aladdin. *N*^6^-hydroxymethyladenosine (hm^6^A), dihydrouridine (D), pseudouridine (Ψ), 2-thiocytidine (s^2^C), 5-hydroxymethylcytidine (hm^5^C), 5-formylcytidine (f^5^C), *N*^2^,*N*^2^-methylguanosine (m^2,2^G), and *N*^1^-methylguanosine (m^1^G) were purchased from TRC-Canada. 2′-O-methyladenosine and *N*^4^-acetylcytidine (ac^4^C) were purchased from Shanghai-Yuanye. *N*^6^-isopentenyladenosine (i^6^A) was obtained from Macklin. 5-methoxyuridine (mo^5^U) was bought from HoweiPharm. *N*^2^-methylguanosine (m^2^G) was purchased from TopScience. 7-methylguanosine was obtained from Sigma. Stock solutions of nucleosides (10–100 mM) were prepared in dimethyl sulfoxide and stored at −20 °C. Mixed standard solutions were prepared by diluting stock solutions with 0.1% formic acid in acetonitrile/water (90/10, *v*/*v*) for hydrophilic interaction liquid chromatography (HILIC).

LC-MS-grade formic acid, water, and acetonitrile were bought from ThermoFisher or Sigma. Reagents for RNA extraction and gel electrophoresis, including cetyltrimethylammonium bromide (CTAB), sodium dodecyl sulfate (SDS), and phenol, were bought from Aladdin or Macklin.

### 2.2. Extraction of Clean tRNA Pools from Coconut Flesh

One gram of coconut endosperm was frozen in liquid nitrogen and ground to powder. Total RNA was isolated with a CTAB-based protocol [28] with modifications for lipid-rich plant samples. Briefly, 1 g powder of coconut flesh (wet weight) was vortexed with 15 mL CTAB lysis buffer (0.1 M Tris-HCl, pH 8.0; 50 mM EDTA, pH 8.0; 1.4 M NaCl; 2% *w*/*v* CTAB; and 2% *w*/*v* PVP-24000 (polyvinylpyrrolidone)), 15 mL phenol, and 3 mL β-mercaptoethanol and heated at 65 °C for 10 min. Next, 16 mL of 1-bromo-3-chloropropane was added while stirring. The mixture was centrifuged at 12,000 rpm for 10 min at 4 °C after cooling on ice for 2 min. The aqueous phase was mixed with an equal volume of acidic phenol and extracted twice with 1-bromo-3-chloropropane to completely remove the protein contaminants. Total RNA was precipitated by adding a 10% volume of 3 M NaOAc (pH 5.5) and three volumes of ethanol, followed by incubation at –80 °C for at least 2 h. The precipitate was collected with centrifugation, washed twice with 70% ethanol, and dried under a vacuum. Total RNA (100 μg) was resolved using 10% urea-PAGE and stained with toluidine blue O. Gels containing tRNA pools (less than 120 nt) were cut and extracted using conventional methods. The integrity of the tRNA pool was confirmed with 10% urea-PAGE.

### 2.3. Enzymatic Digestion of tRNAs

The isolated tRNA pool was digested to release free nucleosides, as previously described [29]. In a buffer containing 50 mM Tris-HCl (pH 8.0), 1 mM MgCl_2_, and 0.1 mg/mL BSA, 10 μg tRNA pool was added with 10 units of benzonase, 0.1 unit of phosphodiesterase I, and 1 unit of alkaline phosphatase. The mixture was incubated at 37 °C for at least 3 h. The enzyme was then removed using a 10 kDa cut-off centrifugal filter tube (Merck). The nucleosides were dried under vacuum and dissolved in an appropriate volume of 90% (*v*/*v*) acetonitrile containing 0.1% formic acid.

### 2.4. Nucleoside Analysis

Nucleosides digested from the tRNA pool of coconut or standard chemicals were subjected to HPLC-HRMS using our established method [30]. Mass spectrometry was conducted on a Uitimate3000-QExactive LC-MS system (Thermo Fisher, Germany). Nucleosides were run in a HILIC separation on a Waters Acquity UPLC BEH amide column (2.1 mm × 150 mm, 1.7 μm) at a flow rate of 0.1 mL/min using the following elution gradient: 0–5 min, 90% B; 5–35 min, 90–40% B; 35–40 min, 40% B; 40–40.1 min, 40–90% B; and 40.1–50 min, 90% B. Mobile phase A was 0.1% (*v*/*v*) formic acid in the water, and mobile phase B was acetonitrile containing 0.1% (*v*/*v*) formic acid. The column temperature was kept at 36 °C, the autosampler was at room temperature, and the injection volume was 1 μL. MS and MS/MS detection used an electrospray ionization (ESI) source with positive ion mode and the following optimized parameters: ion-spray voltage of 6.0 kV, capillary temperature of 150 °C, sheath gas flow rate of 25 L/min, aux gas flow rate of 16 L/min, S-lens RF level of 50.0, and aux gas heater temperature at 350 °C. The MS1 range was set at 200–950 *m*/*z*, and the collision energy of MS2 (NCE) was 0, 100, and 200 V. Xcalibur 4.0 software (Thermo Fisher) was used for the data analysis. Modified nucleosides were identified with the exact mass (mass tolerance < 10 ppm) and retention time of standards.

### 2.5. Oligonucleotide Analysis

In total, 5 μg of coconut’s total tRNA was cut by RNase A or RNase T_1_. Then, oligonucleotides with or without modifications were subjected to HPLC-HRMS using our established method [31]. In the case of mapping pseudouridines, 5 μg of total tRNA that dissolved in 3 μL of Milli-Q water was added to 30 μL of 41% (*v*/*v*) ethanol/1.1 M trimethylammonium acetate (pH 8.6) and 5 μL of acrylonitrile. The mixture was incubated at 70 °C for 2 h to complete pseudouridylation. Cyanoethylated tRNAs were ethanol precipitated, dialyzed, and digested with RNase T_1_. The resulting oligonucleotides were analyzed as above.

### 2.6. Bioinformatic Analysis

The protein sequences of known tRNA-modifying enzymes from *Saccharomyces cerevisiae* (yeast), *E. coli,* and other model microorganisms from the modomics database (https://genesilico.pl/modomics/, accessed on 13 February 2023) were used as query sequences. The annotated proteome of *C. nucifera* L. was obtained from UniProt (https://www.uniprot.org, accessed on 13 February 2023): ID, UP000797356. Blastp was performed with a cut-off value of 1.0 × 10^−6^ for the initial identification of candidate genes. Protein function with domain analysis was manually confirmed on InterPro (https://www.ebi.ac.uk/interpro/, accessed on 13 February 2023) [32], an updated platform from Pfam. The logos and conserved motifs were identified using the MEME online search engine (http://meme-suite.org/tools/meme, accessed on 13 February 2023) [33]. 

All verified tRNA-modifying enzyme genes were mapped to the *C. nucifera* L. genome chromosomes with ID ASM812446v1 of the genome data viewer (https://www.ncbi.nlm.nih.gov/genome/gdv, accessed on 13 February 2023) and the MG2C v2.1 tool (http://mg2c.iask.in/mg2c_v2.1/, accessed on 13 February 2023) [34]. A non-rooted neighborhood joining tree of genes was conducted with MEGA11 software [35], and bootstrap analysis was performed with 2000 iterations. Detailed protein sequence alignments were conducted and visualized with COBALT (https://www.ncbi.nlm.nih.gov/tools/cobalt/cobalt.cgi, accessed on 13 February 2023). 

The tRNAscan-SE 2.0 [25] combined with manual checking found 241 tRNA genes in the genome data of coconut (GCA_008124465.1). Speculated mature tRNA sequences were uploaded in the supplementary materials. The sequence list and raw data of oligonucleotides analysis (RNase A or T_1_) were subjected to RNA Modmapper [26] for preliminary mapping of tRNA modifications.

### 2.7. Mining of Transcriptome Data

Transcriptomic sequencing data of *C. nucifera* L. under high-salinity stress were downloaded from GEO (Gene Expression Omnibus, http://www.ncbi.nlm.nih.gov/gds/, accessed on 13 February 2023), Accession ID: GSE134410. Young coconut plants from two varieties (Hainan Tall and Aromatic Dwarf) were subjected to high-salinity stress. Then, leaf RNA was extracted and sequenced at the following time points: 0 h, 4 h, 6 days, and 10 days after saltwater application [24]. Salt stress added to Hainan Tall (BD) and Aromatic Coconut (XS) plants was performed using irrigation of Murashige and Skoog (MS) culture medium with 200 mM NaCl. Sequencing results (FPKM, the metric fragments per kilobase of feature per million mapped reads) of tRNA-modifying enzyme genes were manually selected with gene ID. Expression levels were assessed using TPM (transcripts per million). Pearson correlation analysis was performed using Prism 8.0.

## 3. Results and Discussion 

### 3.1. Detection of tRNA Modifications with HPLC-HRMS

LC-MS is an effective technique for analyzing nucleosides. Nucleosides possess a wide range of hydrophobicity resulting from the property of a modified moiety. Thus, they can be separated with both reverse phase (RP) and HILIC analytical HPLC columns [36,37]. In addition, in the part of mass spectrometry, triple quadrupole (TQ)-MS and HRMS could be used to identify nucleosides with the multiple reaction monitoring (MRM) mode or precise *m*/*z,* respectively [38].

**Figure 1:**
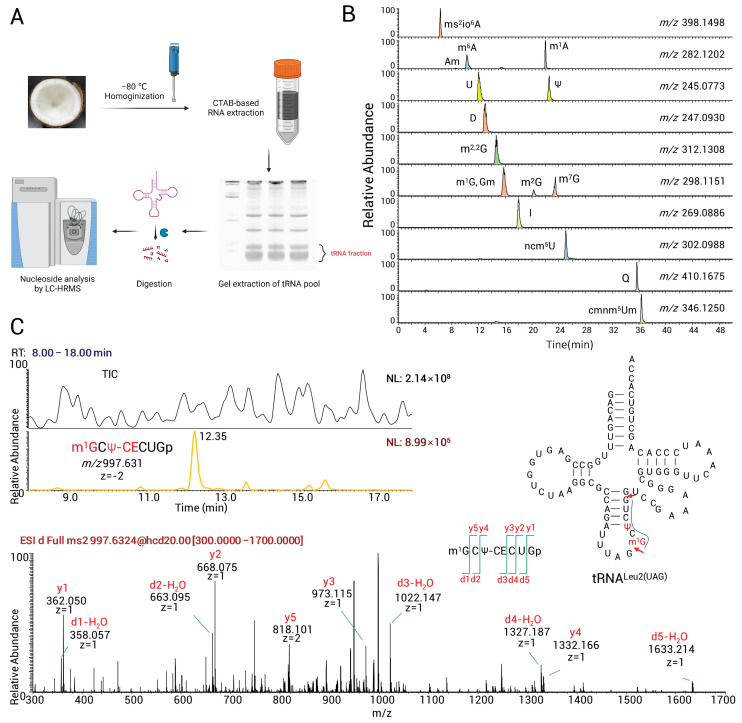
Identification of tRNA modifications with LC-HRMS. (**A**) The tRNAs from the endosperm of coconut (Hainan Tall) were extracted, digested, and subjected to LC-HRMS. (**B**) The detection of 16 species of modified nucleosides is shown. (**C**) An example of mapping tRNA modifications. The RNA fragment [m^1^G]C[Ψ-CE]CUGp was resulted from RNase T_1_ digestion (cleavage at red arrows, upper right panel) and confirmed by exact *m/z* (upper left panel, TIC and XICs). Positions of m^1^G and Ψ were confirmed by CID fragment (lower panel). The fragment was aligned to tRNA^Leu(UAG)^ (upper right panel); thus m^1^G37 and Ψ39 could be mapped.

In our previous studies, the HILIC method resulted in better separation of structural isomers. Meanwhile, precise *m*/*z* combined with standards could prevent the mass cross-talk found in TQ-MS [29,30]. The tRNA pool of the coconut (Hainan Tall) was extracted, digested, and subjected to HILIC-HRMS (Figure 1A). Twenty-five species of tRNA modification were detected and are partially shown in Figure 1B. We listed in Table 1 all modified nucleosides detected using HRMS and the case of whether synthetic standards confirmed them.

### 3.2. Identification of Candidate tRNA-Modifying Enzyme Genes in C. nucifera L.

The tRNA-modifying enzyme candidate genes in the genome of the coconut were identified based on protein sequence homology (cut value: 1.0 × 10^−6^) with modifying enzymes from yeast, *E. coli*, or other model microorganisms [3]. All results are listed in Table 1. The candidate genes annotated as tRNA-modifying enzymes in the uniport coconut proteome (UP000797356) were also added.

In total, 66 candidate genes of 33 modified nucleosides on various positions of tRNA were identified with homologous sequence alignment or annotation. The number of tRNA modification species was more significant than that detected with LC-HRMS (25 species). This was not contradictory, as some tRNA modifications showed tissue-specific expression [39]. Some modified nucleosides, like Ar(p), possess a negative charge, which is not sensitive in the positive ESI mode. Im (2′-O-methylinosine) and ncm^5^U (5-carbamoylmethyluridine) were detected using HRMS, but their modifying enzymes were not found in any organisms. The function of 66 candidate proteins was verified with a domain analysis on InterPro and is listed in Table 2. 

### 3.3. Mapping of Expected tRNA Modifications

**Figure 2:**
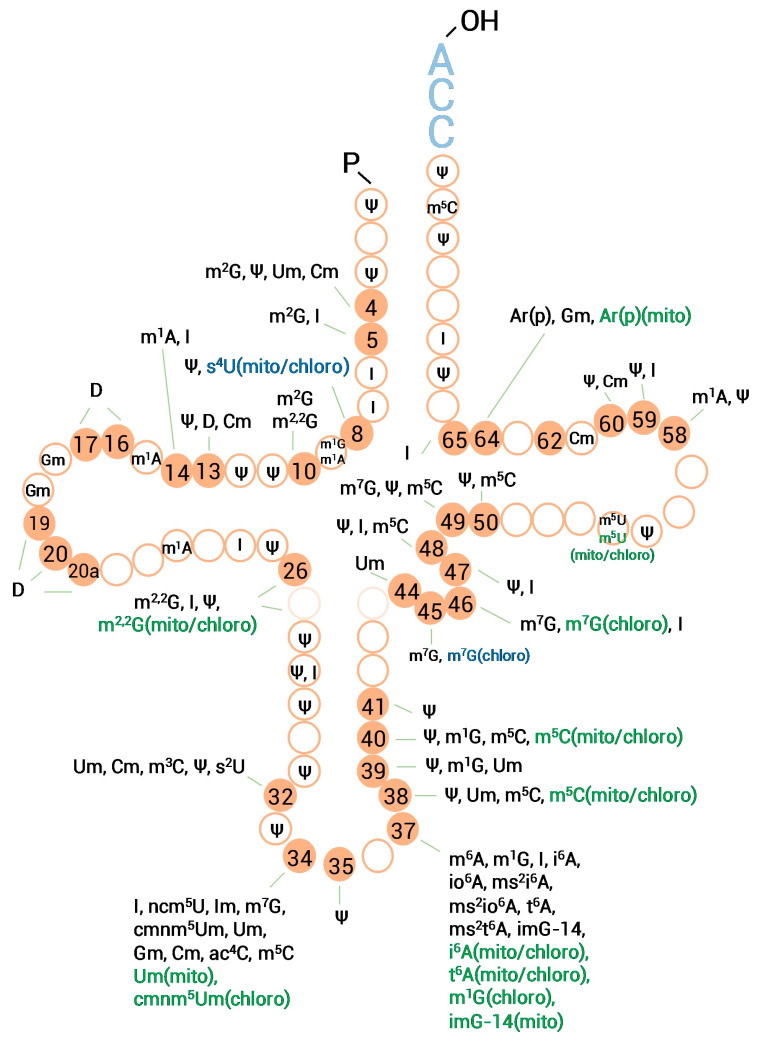
A preliminary landscape of tRNA modifications in *C. nucifera*. Modifications with green or blue colors were speculated as mitochondrial or chloroplastic tRNA modifications. Blue modifications were detected with RNA-MS but rare in eukaryotic tRNAs. Green modifications were affiliated with tRNA-modifying enzymes that were predicted to have organellar translocalization.

**Figure 3:**
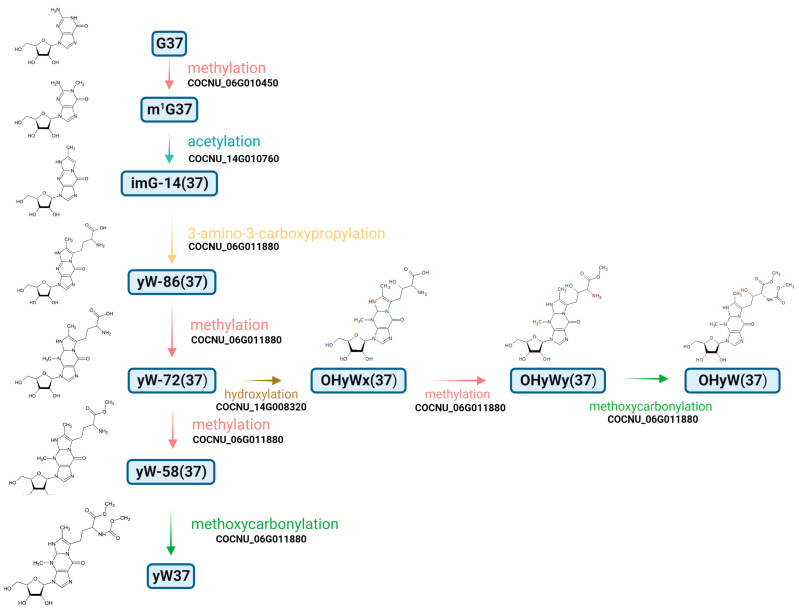
The proposed pathway modified G37 to yW37/OHyW37 in *C. nucifera*.

As described above, the natural or cyanoethylated coconut’s total tRNA was digested and subjected to an oligonucleotide analysis. Combined with the result (MS1 and MS2) of RNase A and RNase T_1_ fragments, the RNA Modmapper mapped a preliminary landscape of tRNA modifications in *C. nucifera* L., as shown in Figure 1C and Figure 2. The coconut’s tRNA was heavily charged. In this map, 105 positions are pointed out, of which positions 34 and 37 were the most frequently modified. Because pseudouridine (ψ) and inosine (I) were installed on multiple sites, we manually checked all unique fragments supporting unreported or rarely reported positions (Table 3 and Table 4). In addition, the modifications Ar(p), imG-14, ms^2^i^6^A, m^3^C, and s^2^U, which were not detected in the nucleoside analysis, could be found and mapped with the oligonucleotide analysis. From the protein blasting results, we could draw a metabolic pathway of candidate enzymes modifying imG-14 to yW or OHyW (Figure 3) [40,41]; however, only imG-14(37) was found. Thus, it hints that the pathway from yW-86 to yW or OHyw is not expressed in coconut endosperm but in other tissues such as leaf and stem tissues. 

Mitochondria and chloroplasts have their own genome, a circular DNA like the genome of eubacteria. Organellar genomes generally encode the partial or full set of tRNAs, rRNAs, and essential proteins of the respiratory or photosynthetic electron transport chains. Organellar tRNAs were modified by enzymes encoded in the host nucleus. These modifying enzymes were usually guided by prepositive mitochondria- or chloroplast-targeting signal peptides [10,42]. Unlike eukaryotic cytoplasmic tRNAs, modifications on organellar tRNA have similar patterns to their bacterial ancestors [43,44]. From an unpublished mitochondrial genome sequence (NC_031696.1) and a chloroplastic genome sequence (NC_022417.1) [45] of the coconut, we mined 24 chloroplastic tRNAs and 24 mitochondrial tRNAs (supplementary materials). These inadequate numbers imply that coconut organelles use some cytoplasmic tRNAs. DNA probe-based isolation of specific tRNAs and the RNA-MS techniques can uncover the complete landscape of coconut tRNA modifications. Here, we provide a preliminary map for further research.

Organellar tRNA modifications can be speculated with subcellular localizations of modifying enzymes, as annotated in Table 1 and added in Figure 2. Also, some detected modified positions that are rare in eukaryotic tRNAs, such as s^4^U8 and m^7^G45, are marked as mitochondrial or chloroplastic tRNA modifications in Figure 2. An early report published evidence showing m^7^G45 of spinach chloroplastic threonine tRNA [46].

### 3.4. Locations of Candidate tRNA-Modifying Enzyme Genes on Coconut Chromosomes

**Figure 4:**
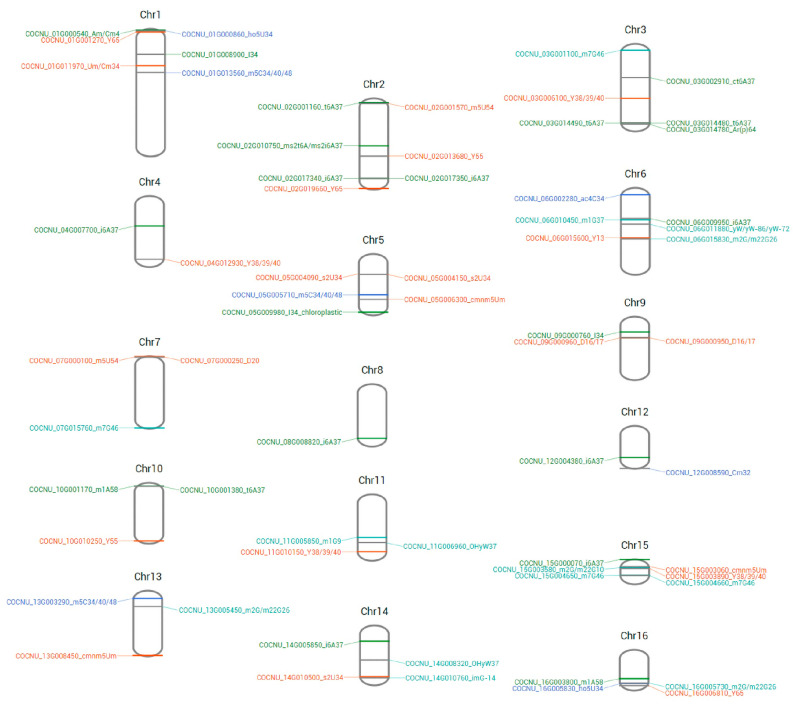
The chromosomal location of the tRNA-modifying enzyme candidate genes of coconut. Modifications on adenosine (A), uridine (U), cytidine (C), and guanosine (G) were distinguished between with colors.

The chromosomal location of the tRNA-modifying enzyme candidate genes of the coconut is shown in Figure 4. The size of each chromosome is listed in Table 5, which can be used to estimate the scale. It was indicated with chromosomal distribution models that certain chromosomes and chromosomal regions had a relatively high distribution of tRNA-modifying enzyme genes. For instance, Chr.4, Chr.7, Chr.8, Chr.9, Chr.10, and Chr.12 contained only one or two tRNA-modifying enzyme genes, respectively. Eleven of sixty-six candidate genes comprise the total, although they occupy a 34.4% sequence of the whole genome. Chr.15 was the shortest chromosome but the one with the highest density of candidate genes. Genes for modifying A and U nucleosides appear evenly on each chromosome, whereas C- and G-modifying genes are limited to specific regions. There are tandem-duplicated gene pairs, including COCNU_02G017340 and COCNU_02G017350 for i^6^A37, COCNU_03G014480 and COCNU_03G014490 for t^6^A37, COCNU_09G000950 and COCNU_09G000960 for D16/17, and COCNU_15G004650 and COCNU_15G004660 for m^7^G46. Highly conserved modifications like t^6^A, i^6^A, Ψ, D, and m^2,2^G have multiple variant genes distributed on different chromosomes. These modifications may ensure their expression under conditional activation or inactivation of certain chromatin regions during development.

### 3.5. i^6^A37-Modifying Enzymes of C. nucifera

**Figure 5:**
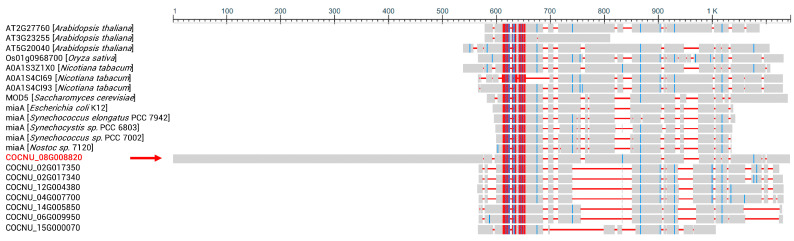
Protein sequence alignment of i^6^A37-modifying enzymes from nine model species and *C. nucifera*. Red indicates highly conserved positions, and blue or gray indicates lower conservation. The red arrow points to an unusual overhanging presequence (575 a.a.) of the translation product COCNU_08G008820.

Eight i^6^A37-modifying enzyme genes can be found in the coconut by aligning with the *E. coli* i^6^A37-modifying enzyme miaA, spreading on seven chromosomes, with the most significant number of variant genes. These eight gene products were aligned with i^6^A37-modifying enzymes of model species, including *E. coli* K12/*Synechocystis* sp. PCC 6803/*Synechococcus* sp. PCC 7002/*Nostoc* sp. 7120 (eubacteria), *S. cerevisiae* (fungus), and *Arabidopsis thaliana*/*Oryza sativa*/*Nicotiana tabacum* (higher plant), using COBALT and clustering to investigate the evolutionary relationships. Highly conserved regions were consistent among procaryotic and eukaryotic creatures, as shown in Figure 5.

**Figure 6:**
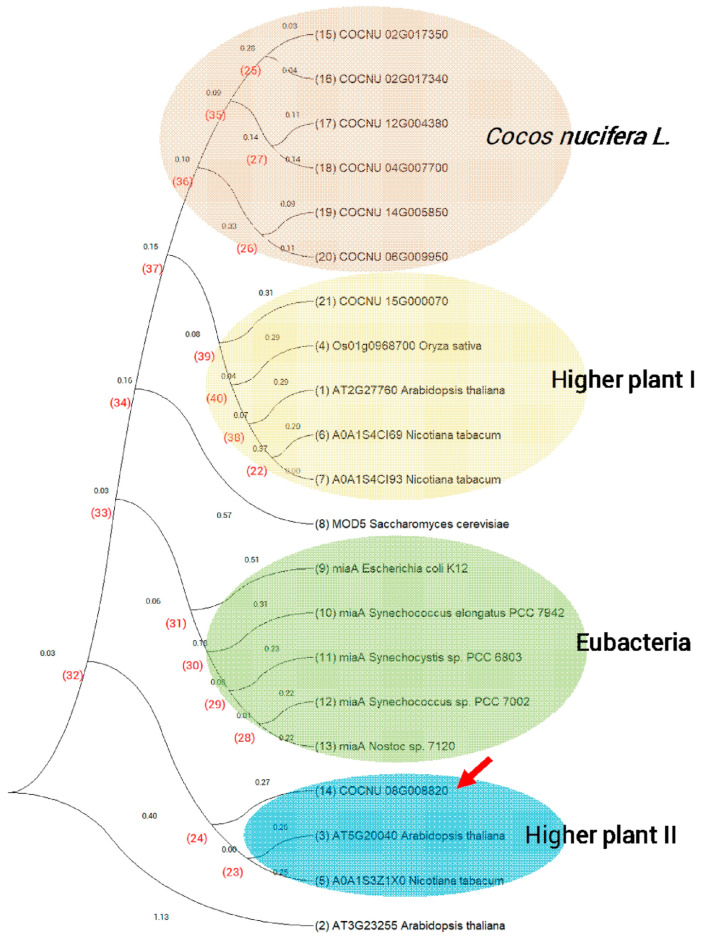
Curved neighbor-joining (N–J) tree of i^6^A37-modifying enzyme candidate genes. Supporting values from the bootstrap analysis are shown for each branch. Six genes of *C. nucifera* were identified in branches different from higher plants.

**Figure 7:**
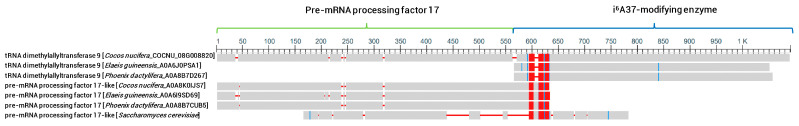
Protein sequence alignment of COCNU_08G008820 (coconut) with i^6^A37-modifying enzymes and pre-mRNA processing factor 17 in palm plants and yeast.

Interestingly, the variant gene COCNU_08G008820 has a protein product possessing an unusually long overhanging presequence (1–575 a.a.). These i^6^A37-modifying enzymes clustered consistently with their categories by building a neighbor-joining (N-J) evolutionary tree (Figure 6). Meanwhile, overhanging-presequence (1–575 a.a.) high conservation with pre-mRNA processing factor 17 of palm plants and yeast was shown with the COCNU_08G008820 product (Figure 7). It looks like a combination of the i^6^A37-modifying enzyme and pre-mRNA processing factor 17. It is unique in the coconut and not similar to close species, such as *Elaeis guineensis* (oil palm) and *Phoenix dactylifera* (date palm). Pre-mRNA processing factor 17, also named CDC40p in yeast, is required to regulate both DNA replication and mitotic spindle formation [47]. It may function as part of a spliceosome and work in the second step of pre-mRNA splicing. COCNU_08G008820 hinders that (1) i^6^A37 modification may happen during pre-tRNA splicing and (2) i^6^A may exist on mRNA. In either case, this is a unique phenomenon in the only plant of the genus *Cocos*. 

### 3.6. The Functional Domain Motif among tRNA Methyltransferases (MTases)

**Figure 8:**
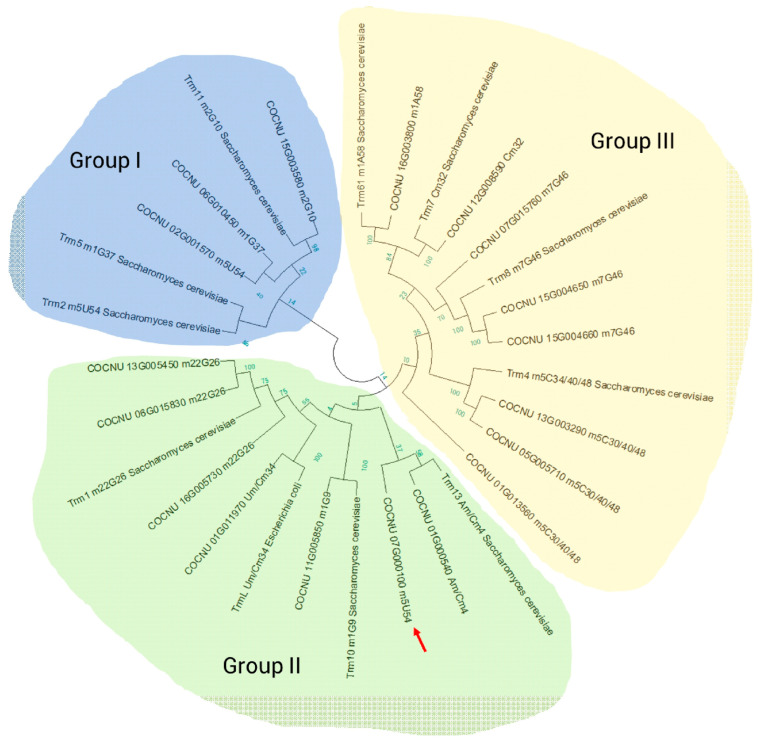
Circular neighbor-joining (N–J) tree of candidate coconut MTase genes. Each branch displays supporting values from the bootstrap analysis. The three protein groups clustered together are marked as follows: group I, shades of blue; group II, shades of green; and group III, shades of yellow.

Methylation is the most common and abundant type of hundreds of modified nucleosides in tRNA molecules. Nucleoside methylation at the base or 2′-O position is catalyzed by tRNA MTases [48,49]. The tRNA MTases in *S. cerevisiae* are mainly AdoMet-dependent methyltransferases located in the RFM superfamily or the SPOUT superfamily [50,51]. Thus, an alternative approach to classifying AdoMet-dependent MTases was proposed, using the catalytic domain as a criterion for functional annotation [52]. Yeasty tRNA MTases are considered a good reference for the study of tRNA MTases in higher plants. Although some tRNA transferases have been reported in Arabidopsis [53,54,55], little is known about them in monocots. We identified 18 MTases in the coconut using alignment with known MTases in yeast and *E. coli* (Table 1 and Table 2). The neighbor-joining (N-J) tree clustered three groups of MTases (Figure 8): (1) group I for methylation on U and G; (2) group II for methylation on A, U, C, and G; and (3) group III for conserved methylation on A, C, and G. Although common features of m^5^U- and m^5^C-modifying enzymes were shown in a previous study, a tentative evolutionary route of 5-methylpyrimidine MTases was suggested [56]. MTases have been suggested to have evolved independently in the coconut. G modification enzymes were split into three groups: m^1^G37 and m^2^G10 (group I), m^2,2^G26 and m^1^G9 (group II), and m^7^G46 (group III). This is consistent with the alignment results in rice and *Arabidopsis* [57]. An unusual base methylation enzyme gene, COCNU_07G000100 (shown in Figure 8), was clustered with the 2′-O methylation enzyme. It may have dual catalytic activity at the methylation site.

**Figure 9:**
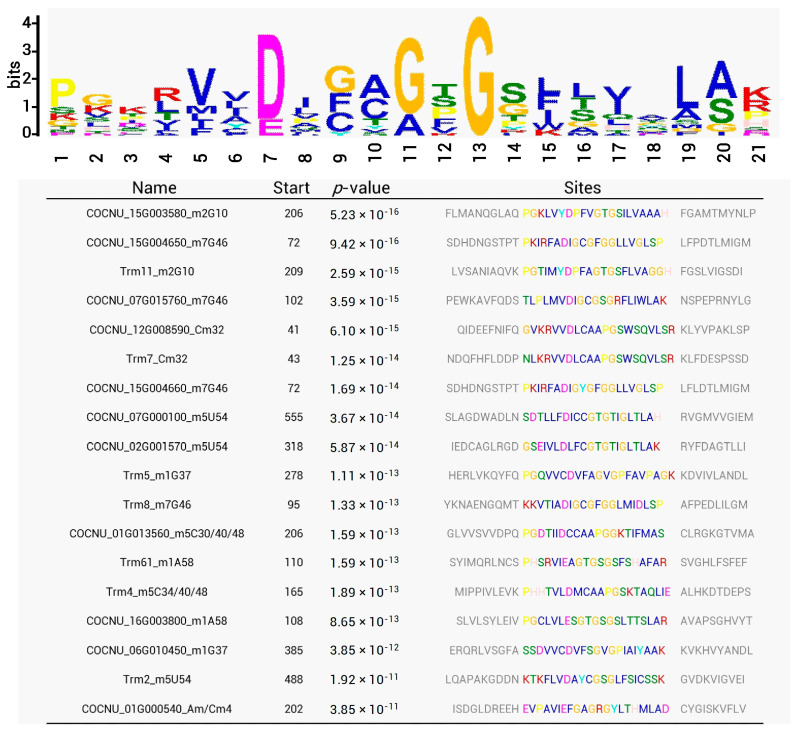
Conserved motif analysis of coconut tRNA MTase candidate genes. The X-axis represents the position of each residue in the identified pattern, and the Y-axis represents the value of the number of bits. Detailed information on the motif sequence of each protein (e.g., name, starting amino acid, *p*-value, and motif sequence) is shown in the table below.

A conserved motif analysis using the MEME program revealed the presence of conserved residues among 11 coconut and 7 yeast tRNA MTases for all nucleoside (A, U, C, and G) methylation (Figure 9). These 18 tRNA MTases shared significant conservation of glycine (G) residues flanked by the consensus motif “VNDNGN(G/A)NG”. It is suggested with strong conservation that the motif, including the G residue, is most likely involved in the structures necessary for tRNA binding or catalysis.

### 3.7. Ar(p)64 Overexpression under High-Salinity Stress

**Figure 10:**
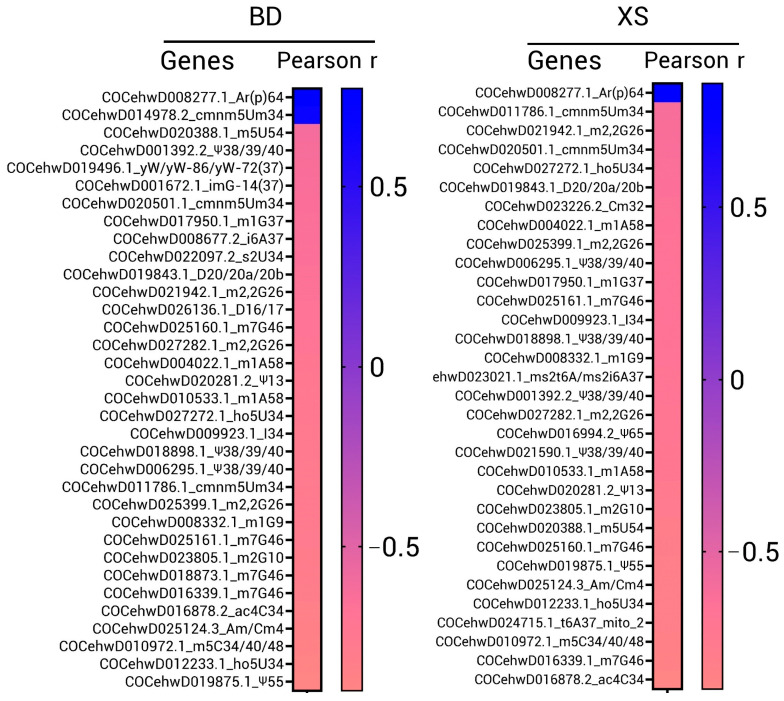
Pearson correlation analysis of the expression of tRNA-modifying enzyme genes and high-salinity stress. The TPM of each gene with salt treatment time (e.g., 0 h, 4 h, 6 d, 10 d) was analyzed, and a cut-off value was set as *p* < 0.05. The Pearson correlation coefficient (r) with significantly relative genes is shown.

BD and XS plants were irrigated with a high-salt medium, and the transcriptome data (leaf) were collected at 0 h, 4 h, 6 days, and 10 days. A Pearson correlation analysis of the expression of tRNA-modifying enzyme candidate genes under stress tolerance was constructed. As shown in Figure 10, most candidate genes are negative relative to high-salinity stress, which is an unfavorable condition for general plant growth. The downregulation of tRNA-modifying enzymes could impair the stability and function of tRNAs. However, high positive relativities of the Ar(p)64-modifying enzyme with high salt were found in both the Hainan Tall (BD) and Aromatic Coconut (XS) variant strains. Ar(p) is a specific modification at position 64 of tRNA^Met(i)^. 5′-phosphoribosyl-1′-pyrophosphate was used as the phosphate donor. After modification, tRNA^Met(i)^ was prevented from participating in the elongation step of protein synthesis. As most modifications were impaired with high salt, the coconut appears to enhance the quality control of the translation process against adverse environmental conditions.

## 4. Conclusions

While nucleoside analogs can be used as drugs and food flavor additives, the physiological function of naturally modified nucleosides (>160 species identified so far) from food intake is mainly unknown. Here, we surveyed tRNA modifications (tRNAs are rich in post-transcriptional modification and are spontaneously degraded to modified nucleosides) and modifying enzymes in the tropical food of the coconut as a template. In total, 33 modified nucleosides on 46 positions of coconut tRNAs were expected. The gene COCNU_08G008820 encodes protein-containing sequences homologous to both pre-mRNA processing factor 17 and the i^6^A37-modifying enzyme miaA. It may be unique and worthy of testing. A conserved motif could be found in 11 MTases of the coconut, which points to a conjunct evolutionary path. The candidate enzyme-modifying Ar(p)64 on tRNA^Met(i)^ was upregulated under high-salinity stress, while most other modifying enzymes were downregulated. As Ar(p)64 is a negatively charged nucleoside that cannot be detected easily with the general positive ESI method of LC-MS, its function can be further confirmed in plants. The change in the expression pattern of tRNA-modifying enzyme genes also leads us to consider the dynamic absorption of naturally modified nucleosides with intake from foods of different seasons and production places. We hope this survey can help advance research on tRNA modification, scientific studies of the coconut, and the safety and nutritional value of naturally modified nucleosides.

## Figures and Tables

**Table 1:** tRNA modifications and modifying enzymes identified with LC-HRMS and homologous protein sequence alignment in *C. nucifera* L.

tRNA Modification	*m*/*z* Value (z = +1)	Found in HRMS	Confirmed by Synthetic Standards	Found in Positions of Coconut tRNA	Homologous Modifying Enzyme	*C. nucifera* L. Candidate Gene ORF Name	*C. nucifera* L. Candidate Gene_ID	Uniprot Protein Name	E-Value	Subcellular Localization Prediction with WoLF PSORT
I	269.0886	√	√	5–7, 14, 24, 28, 34, 37, 46–48, 59, 65, 67	TadA (*Arabidopsis thaliana*)	*COCNU_05G009980*	*COCehwD004402.1*	tRNA(Adenine(34)) deaminase, chloroplastic	1.38 × 10^−82^	nucleus
						*COCNU_09G000760*	*COCehwD000874.2*	tRNA-specific adenosine deaminase TAD2	3.15 × 10^−15^	nucleus
						*COCNU_01G008900*	*COCehwD009923.1*	tRNA-specific adenosine deaminase TAD3	9.00 × 10^−8^	nucleus
					Tad1 (*Saccharomyces cerevisiae*)	*COCNU_09G007590*	*COCehwD021876.1*	polyamine-modulated factor 1-binding protein 1	8.00 × 10^−17^	nucleus
m^6^A	282.1202	√	√	37	TrmM (*Escherichia coli*)	*N.D.*				
Am	282.1202	√	√	4	Trm13 (*Saccharomyces cerevisiae*)	*COCNU_01G000540*	*COCehwD025124.3*	tRNA:m(4)X modification enzyme TRM13	5.49 × 10^−15^	cytoplasm
m^1^A	282.1202	√	√	9, 14, 15, 22, 58	Trm61 (*Saccharomyces cerevisiae*)	*COCNU_16G003800*	*COCehwD004022.1*	tRNA (Adenine(58)-N(1))-methyltransferase catalytic subunit TRMT61A	2.47 × 10^−58^	cytoplasm
					Trm6 (*Saccharomyces cerevisiae*)	*COCNU_10G001170*	*COCehwD010533.1*	tRNA (Adenine(58)-N(1))-methyltransferase non-catalytic subunit trm6	5.75 × 10^−17^	nucleus
Im	283.1042	√	√	64	not found yet					
i^6^A	336.1672	√	√	37	MiaA (*Escherichia coli*)	*COCNU_08G008820*	*COCehwD008677.2*	Putative tRNA dimethylallyltransferase 9	4.06 × 10^−36^	nucleus
						*COCNU_02G017350*	*COCehwD026185.1*	Adenylate isopentenyltransferase 5, chloroplastic	4.18 × 10^−19^	cytoplasm
						*COCNU_02G017340*	*COCehwD026184.1*	Adenylate isopentenyltransferase 5, chloroplastic	1.28 × 10^−18^	cytoplasm
						*COCNU_12G004380*	*COCehwD019007.1*	Adenylate isopentenyltransferase 5, chloroplastic	3.89 × 10^−16^	cytoplasm
						*COCNU_04G007700*	*COCehwD012340.1*	Adenylate isopentenyltransferase 5, chloroplastic	4.74 × 10^−15^	chloroplast
						*COCNU_14G005850*	*COCehwD012201.1*	Adenylate isopentenyltransferase 1, chloroplastic	1.80 × 10^−14^	mitochondrion
						*COCNU_06G009950*	*COCehwD002451.1*	Adenylate isopentenyltransferase 1, chloroplastic	1.03 × 10^−11^	chloroplast
						*COCNU_15G000070*	*COCehwD005848.1*	Putative adenylate isopentenyltransferase 5, chloroplastic	3.54 × 10^−9^	cytoplasm
io^6^A	352.1621	√	×	37	MiaE (*Salmonella typhimurium*)	N.D.				
ms^2^io^6^A	398.1498	√	×	37	MiaE (*Salmonella typhimurium*)	N.D.				
t^6^A	413.1421	√	×	37	TsaD (*Escherichia coli*)	*COCNU_10G001380*	*COCehwD010512.1*	Putative tRNA N6-adenosine threonylcarbamoyltransferase, mitochondrial	4.33 × 10^−67^	chloroplast
						*COCNU_02G001160*	*COCehwD024715.1*	Putative tRNA N6-adenosine threonylcarbamoyltransferase, mitochondrial	1.14 × 10^−16^	cytoplasm
						*COCNU_03G014480*	*COCehwD015914.1*	Putative tRNA N6-adenosine threonylcarbamoyltransferase	1.10 × 10^−8^	nucleus
						*COCNU_03G014490*	*COCehwD015913.1*	Putative tRNA N6-adenosine threonylcarbamoyltransferase	annotated	chloroplast
ct^6^A	395.1315	×	×	37	TcdA (*Escherichia coli*)	*COCNU_03G002910*	*COCehwD019353.1*	Putative tRNA threonylcarbamoyladenosine dehydratase	annotated	cytoplasm
ms^2^t^6^A	459.1298	√	×	37	MtaB (*Bacillus subtilis*)	*COCNU_02G010750*	*COCehwD023021.1*	Putative threonylcarbamoyladenosine tRNA methylthiotransferase	5.96 × 10^−28^	nucleus
Ψ	245.0773	√	√	1, 3, 4, 8, 11, 12, 25, 27–29, 31–33, 35, 39–41, 47–50, 55, 59, 60, 67, 71, 73	TruA (*Escherichia coli*)	*COCNU_15G003890*	*COCehwD006295.1*	tRNA pseudouridine synthase	1.25 × 10^−19^	nucleus
						*COCNU_03G006100*	*COCehwD018898.1*	tRNA pseudouridine(38/39) synthase	6.30 × 10^−15^	nucleus
						*COCNU_11G010150*	*COCehwD001392.2*	tRNA pseudouridine synthase	4.21 × 10^−11^	cytoplasm
						*COCNU_04G012930*	*COCehwD021590.1*	Putative tRNA pseudouridine synthase	annotated	chloroplast
					TruB (*Escherichia coli*)	*COCNU_10G010250*	*COCehwD019875.1*	H/ACA ribonucleoprotein complex subunit 4	3.10 × 10^−15^	cytoplasm
						*COCNU_02G013680*	*COCehwD018401.1*	Putative tRNA pseudouridine synthase 1	1.45 × 10^−14^	chloroplast
					TruC (*Escherichia coli*)	*COCNU_16G006810*	*COCehwD024938.1*	RNA pseudouridine synthase 5	1.23 × 10^−13^	mitochondrion
						*COCNU_02G019660*	*COCehwD006418.1*	RNA pseudouridine synthase 3, mitochondrial	3.63 × 10^−10^	chloroplast_mitochondrion
						*COCNU_01G001270*	*COCehwD016994.2*	Putative serine/threonine–protein phosphatase 4 regulatory subunit 3	6.35 × 10^−8^	nucleus
					TruD (*Escherichia coli*)	*COCNU_06G015600*	*COCehwD020281.2*	Putative tRNA pseudouridine13 synthase	annotated	nucleus
D	247.0930	√	√	16, 17, 19, 20, 20a	Dus1 (*Saccharomyces cerevisiae*)	*COCNU_09G000950*	*COCehwD026136.1*	Putative tRNA-dihydrouridine(16/17) synthase [NAD(P)(+)]-like	4.16 × 10^−38^	cytoplasm
						*COCNU_09G000960*	*COCehwD026137.1*	Putative tRNA-dihydrouridine(16/17) synthase [NAD(P)(+)]-like	8.32 × 10^−14^	cytoplasm
					Dus2, Dus4 (*Saccharomyces cerevisiae*)	*COCNU_07G000250*	*COCehwD019843.1*	Putative tRNA-dihydrouridine(20) synthase [NAD(P)]-like	1.70 × 10^−16^	chloroplast
Um	259.0930	√	√	4, 32, 34, 38, 39, 44	TrmL (*Escherichia coli*)	*COCNU_01G011970*	*COCehwD008780.1*	MADS5	9.48 × 10^−37^	mitochondrion
m^5^U	259.0930	√	√	54	Trm2 (*Saccharomyces cerevisiae*)	*COCNU_02G001570*	*COCehwD004165.1*	Putative RNA methyltransferase	6.85 × 10^−20^	chloroplast_mitochondrion
						*COCNU_07G000100*	*COCehwD020388.1*	Zinc finger CCCH domain-containing protein 24	7.66 × 10^−12^	nucleus
ncm^5^U	302.0988	√	×	34	not found yet					
cmnm^5^Um	346.1250	√	×	34	MnmE (*Escherichia coli*)	*COCNU_05G006300*	*COCehwD014978.2*	tRNA modification GTPase MnmE	4.16 × 10^−72^	chloroplast
						*COCNU_13G008450*	*COCehwD011786.1*	Uncharacterized protein	2.83 × 10^−13^	cytoplasm
					MnmG (*Escherichia coli*)	*COCNU_15G003060*	*COCehwD020501.1*	tRNA uridine 5-carboxymethylaminomethyl modification enzyme MnmG	4.00 × 10^−167^	chloroplast
Cm	258.1090	√	√	4, 13, 32, 34, 60	Trm7 (*Saccharomyces cerevisiae*)	*COCNU_12G008590*	*COCehwD023226.2*	Putative sulfoquinovosyl transferase SQD2	2.98 × 10^−74^	nucleus
					Trm13 (*Saccharomyces cerevisiae*)	*COCNU_01G000540*	*COCehwD025124.3*	tRNA:m(4)X modification enzyme TRM13	5.49 × 10^−15^	cytoplasm
m^5^C	258.1090	√	√	11, 38, 40, 48–50, 72	Trm4 (*Saccharomyces cerevisiae*)	*COCNU_13G003290*	*COCehwD010972.1*	tRNA (Cytosine(34)-C(5))-methyltransferase	4.49 × 10^−93^	mitochondrion
						*COCNU_05G005710*	*COCehwD013570.1*	Putative tRNA (Cytosine(34)-C(5))-methyltransferase	2.06 × 10^−24^	chloroplast
						*COCNU_01G013560*	*COCehwD011889.1*	Uncharacterized protein	6.06 × 10^−18^	chloroplast
ho^5^U	261.0717	√	√	34	TrhO (*Escherichia coli*)	*COCNU_16G005830*	*COCehwD027272.1*	Rhodanese-like domain-containing protein 7	1.36 × 10^−29^	chloroplast
						*COCNU_01G000860*	*COCehwD012233.1*	Rhodanese-like domain-containing protein 6	2.15 × 10^−13^	nucleus
ac^4^C	286.1039	√	√	34	TmcA (*Escherichia coli*)	*COCNU_06G002280*	*COCehwD016878.2*	Putative RNA cytidine acetyltransferase 1	1.26 × 10^−18^	cytoplasm
m^1^G	298.1151	√	√	9, 37, 39, 40	Trm5 (*Saccharomyces cerevisiae*)	*COCNU_06G010450*	*COCehwD017950.1*	tRNA (Guanine(37)-N1)-methyltransferase 1	2.60 × 10^−52^	chloroplast
					Trm10 (*Saccharomyces cerevisiae*)	*COCNU_11G005850*	*COCehwD008332.1*	tRNA (Guanine(9)-N1)-methyltransferase	2.04 × 10^−27^	cytoplasm
m^7^G	298.1151	√	√	34, 45, 46, 47, 49	Trm8 (*Saccharomyces cerevisiae*)	*COCNU_15G004650*	*COCehwD025160.1*	tRNA (Guanine-N(7)-)-methyltransferase	4.86 × 10^−95^	chloroplast
						*COCNU_15G004660*	*COCehwD025161.1*	Putative tRNA (Guanine-N(7)-)-methyltransferase	8.31 × 10^−29^	nucleus
						*COCNU_07G015760*	*COCehwD018873.1*	Putative tRNA (Guanine-N(7)-)-methyltransferase	1.02 × 10^−8^	chloroplast
					Trm82 (*Saccharomyces cerevisiae*)	*COCNU_03G001100*	*COCehwD016339.1*	tRNA (Guanine-N(7)-)-methyltransferase non-catalytic subunit wdr4	1.14 × 10^−14^	cytoplasm
m^2^G	298.1151	√	√	4, 5, 10	Trm11 (*Saccharomyces cerevisiae*)	*COCNU_15G003580*	*COCehwD023805.1*	tRNA (Guanine(10)-N2)-methyltransferase	7.49 × 10^−47^	cytoplasm
m^2,2^G	312.1308	√	√	26, 27	Trm1 (*Saccharomyces cerevisiae*)	*COCNU_06G015830*	*COCehwD021942.1*	Putative tRNA (Guanine(26)-N(2))-dimethyltransferase 1	1.00 × 10^−75^	nucleus
						*COCNU_16G005730*	*COCehwD027282.1*	Putative tRNA (Guanine(26)-N(2))-dimethyltransferase	6.00 × 10^−13^	chloroplast
						*COCNU_13G005450*	*COCehwD025399.1*	tRNA (Guanine(26)-N(2))-dimethyltransferase	1.00 × 10^−25^	mitochondrion
OHyW	525.1945	√	×	37	hTYW5 (*Homo sapiens*)	*COCNU_11G006960*	*COCehwD022435.1*	Lysine-specific demethylase JMJ30	1.61 × 10^−15^	chloroplast
						*COCNU_14G008320*	*COCehwD002793.1*	Putative tRNA wybutosine-synthesizing protein 5	annotated	cytoplasm
imG-14	322.1151	×	×	37	TYW1 (*Saccharomyces cerevisiae*)	*COCNU_14G010760*	*COCehwD001672.1*	Putative S-adenosyl-L-methionine-dependent tRNA 4-demethylwyosine synthase-like	annotated	mitochondrion
yW	509.1996	×	×	37	TYW2/3/4 (*Saccharomyces cerevisiae*)	*COCNU_06G011880*	*COCehwD019496.1*	Putative tRNA wybutosine-synthesizing protein 2/3/4	annotated	nucleus
yW-86	423.1628	×	×	37	TYW2/3/4 (*Saccharomyces cerevisiae*)					
yW-72	437.1784	×	×	37	TYW2/3/4 (*Saccharomyces cerevisiae*)					
yW-58	451.1941	×	×	37	TYW2/3/4 (*Saccharomyces cerevisiae*)					
Ar(p)	-	×	×	64	Rit1 (*Saccharomyces cerevisiae*)	*COCNU_03G014780*	*COCehwD008277.1*	tRNA A64-2’-O-ribosylphosphate transferase	annotated	chloroplast
ms^2^i^6^A	382.1549	×	×	37	MiaB (*Escherichia coli*)	*COCNU_02G010750*	*COCehwD023021.1*	Putative threonylcarbamoyladenosine tRNA methylthiotransferase	6.94 × 10^−27^	nucleus
s^2^U	261.0545	×	×	34	MnmA (*Escherichia coli*)	*COCNU_05G004150*	*COCehwD022097.2*	Putative tRNA-specific 2-thiouridylase MnmA	1.67 × 10^−24^	nucleus
						*COCNU_14G010500*	*COCehwD008162.1*	Cytoplasmic tRNA 2-thiolation protein 2	annotated	nucleus
						*COCNU_05G004090*	*COCehwD027867.1*	Uncharacterized protein	3.29 × 10^−7^	nucleus

Note: “√” means “yes” and “×” means “no”.

**Table 2:** InterPro (Pfam) domain analysis of candidate tRNA modifying enzyme genes in *C. nucifera* L.

tRNA Modification	Gene	Protein Family	Biological Process	Molecular Function
I34	*COCNU_05G009980*	tRNA-specific adenosine deaminase (IPR028883)	tRNA wobble adenosine to inosine editing (GO:0002100)	catalytic activity (GO:0003824) tRNA-specific adenosine deaminase activity (GO:0008251)
	*COCNU_09G000760*	None predicted	None	hydrolase activity (GO:0016787) zinc ion binding (GO:0008270) catalytic activity (GO:0003824)
	*COCNU_01G008900*	None predicted	None	catalytic activity (GO:0003824)
Am/Cm4	*COCNU_01G000540*	tRNA:m(4)X modification enzyme Trm13 (IPR039044)	tRNA methylation (GO:0030488) tRNA processing (GO:0008033)	tRNA 2’-O-methyltransferase activity (GO:0106050) methyltransferase activity (GO:0008168)
m^1^A58	*COCNU_16G003800*	tRNA (1-methyladenosine) methyltransferase catalytic subunit Gcd14 (IPR014816)	tRNA methylation (GO:0030488)	tRNA (adenine-N1-)-methyltransferase activity (GO:0016429)
	*COCNU_10G001170*	tRNA (adenine(58)-N(1))-methyltransferase non-catalytic subunit TRM6 (IPR017423)	tRNA methylation (GO:0030488)	none
i^6^A37	*COCNU_08G008820*	Dimethylallyltransferase (IPR039657) IPP transferase (IPR018022) Pre-mRNA-processing factor 17 (IPR032847)	tRNA processing (GO:0008033) mRNA splicing, via spliceosome (GO:0000398)	protein binding (GO:0005515)
	*COCNU_02G017350*	Dimethylallyltransferase (IPR039657)	No GO Terms	no GO terms
	*COCNU_02G017340*	Dimethylallyltransferase (IPR039657)	No GO Terms	no GO terms
	*COCNU_12G004380*	Dimethylallyltransferase (IPR039657)	No GO Terms	no GO terms
	*COCNU_04G007700*	Dimethylallyltransferase (IPR039657)	No GO Terms	no GO terms
	*COCNU_14G005850*	Dimethylallyltransferase (IPR039657) IPP transferase (IPR018022)	tRNA processing (GO:0008033)	none
	*COCNU_06G009950*	Dimethylallyltransferase (IPR039657)	No GO Terms	no GO terms
	*COCNU_15G000070*	Dimethylallyltransferase (IPR039657)	No GO Terms	no GO terms
t^6^A37	*COCNU_10G001380*	Kae1/TsaD family (IPR017861) tRNA N6-adenosine threonylcarbamoyltransferase, TsaD (IPR022450)	tRNA threonylcarbamoyladenosine modification (GO:0002949)	none
	*COCNU_02G001160*	Kae1/TsaD family (IPR017861) tRNA N6-adenosine threonylcarbamoyltransferase Kae1, archaea and eukaryote (IPR034680)	tRNA threonylcarbamoyladenosine modification (GO:0002949)	none
	*COCNU_03G014480*	Kae1/TsaD family (IPR017861) tRNA N6-adenosine threonylcarbamoyltransferase Kae1, archaea and eukaryote (IPR034680)	tRNA threonylcarbamoyladenosine modification (GO:0002949)	none
	*COCNU_03G014490*	Kae1/TsaD family (IPR017861) tRNA N6-adenosine threonylcarbamoyltransferase Kae1, archaea and eukaryote (IPR034680)	tRNA threonylcarbamoyladenosine modification (GO:0002949)	none
ct^6^A37	*COCNU_03G002910*	ThiF/MoeB/HesA family (IPR045886)	None	ubiquitin-like modifier activating enzyme activity (GO:0008641)
ms^2^t^6^A37	*COCNU_02G010750*	Methylthiotransferase (IPR005839) MiaB-like tRNA modifying enzyme, archaeal-type (IPR006466)	tRNA methylthiolation (GO:0035600)	catalytic activity (GO:0003824) iron-sulfur cluster binding (GO:0051536) N6-threonylcarbomyladenosine methylthiotransferase activity (GO:0035598) methylthiotransferase activity (GO:0035596) 4-iron, 4-sulfur cluster binding (GO:0051539)
Y38/39/40	*COCNU_15G003890*	Pseudouridine synthase I, TruA (IPR001406)	RNA modification (GO:0009451) Pseudouridine synthesis (GO:0001522)	RNA binding (GO:0003723) pseudouridine synthase activity (GO:0009982)
	*COCNU_03G006100*	Pseudouridine synthase I, TruA (IPR001406) Pseudouridine synthase Pus3-like (IPR041707)	Pseudouridine synthesis (GO:0001522) RNA modification (GO:0009451)	RNA binding (GO:0003723) pseudouridine synthase activity (GO:0009982)
	*COCNU_11G010150*	Pseudouridine synthase I, TruA (IPR001406)	Pseudouridine synthesis (GO:0001522) RNA modification (GO:0009451)	pseudouridine synthase activity (GO:0009982) RNA binding (GO:0003723)
	*COCNU_04G012930*	Pseudouridine synthase I, TruA (IPR001406)	Pseudouridine synthesis (GO:0001522) RNA modification (GO:0009451)	RNA binding (GO:0003723) pseudouridine synthase activity (GO:0009982)
Y55	*COCNU_10G010250*	tRNA pseudouridine synthase B family (IPR004802)	Pseudouridine synthesis (GO:0001522) RNA modification (GO:0009451) RNA processing (GO:0006396)	RNA binding (GO:0003723) pseudouridine synthase activity (GO:0009982)
	*COCNU_02G013680*	tRNA pseudouridine synthase II, TruB (IPR014780)	RNA modification (GO:0009451) Pseudouridine synthesis (GO:0001522) RNA processing (GO:0006396)	pseudouridine synthase activity (GO:0009982) RNA binding (GO:0003723)
Y65	*COCNU_16G006810*	None predicted	RNA modification (GO:0009451) Pseudouridine synthesis (GO:0001522) RNA modification (GO:0009451)	pseudouridine synthase activity (GO:0009982) RNA binding (GO:0003723)
	*COCNU_02G019660*	None predicted	RNA modification (GO:0009451) Pseudouridine synthesis (GO:0001522)	RNA binding (GO:0003723) pseudouridine synthase activity (GO:0009982)
	*COCNU_01G001270*	None predicted	RNA modification (GO:0009451) Pseudouridine synthesis (GO:0001522)	RNA binding (GO:0003723) pseudouridine synthase activity (GO:0009982)
Y13	*COCNU_06G015600*	Pseudouridine synthase, TruD (IPR001656)	RNA modification (GO:0009451) Pseudouridine synthesis (GO:0001522)	pseudouridine synthase activity (GO:0009982) RNA binding (GO:0003723)
D16/17	*COCNU_09G000950*	None predicted	No GO Terms	no GO terms
	*COCNU_09G000960*	None predicted	No GO Terms	no GO terms
D20/20a/20b	*COCNU_07G000250*	None predicted	tRNA processing (GO:0008033)	tRNA dihydrouridine synthase activity (GO:0017150) flavin adenine dinucleotide binding (GO:0050660)
Um	*COCNU_01G011970*	tRNA (cytidine/uridine-2’-O-)-methyltransferase (IPR016914)	RNA methylation (GO:0001510) RNA processing (GO:0006396)	methyltransferase activity (GO:0008168) RNA binding (GO:0003723) RNA methyltransferase activity (GO:0008173)
m^5^U54	*COCNU_02G001570*	(Uracil-5)-methyltransferase family (IPR010280)	RNA processing (GO:0006396)	RNA methyltransferase activity (GO:0008173)
	*COCNU_07G000100*	(Uracil-5)-methyltransferase family (IPR010280) tRNA (uracil(54)-C(5))-methyltransferase, metazoa type (IPR045850)	RNA processing (GO:0006396)	RNA methyltransferase activity (GO:0008173) metal ion binding (GO:0046872) nucleic acid binding (GO:0003676) RNA binding (GO:0003723)
cmnm^5^Um34	*COCNU_05G006300*	tRNA modification GTPase MnmE (IPR004520)	tRNA modification (GO:0006400)	GTP binding (GO:0005525) GTPase activity (GO:0003924) protein binding (GO:0005515)
	*COCNU_13G008450*	GTP-binding protein EngA (IPR016484)	None	GTP binding (GO:0005525)
	*COCNU_15G003060*	tRNA uridine 5-carboxymethylaminomethyl modification enzyme MnmG-related (IPR002218) tRNA uridine 5-carboxymethylaminomethyl modification enzyme MnmG (IPR004416)	tRNA wobble uridine modification (GO:0002098) tRNA processing (GO:0008033)	flavin adenine dinucleotide binding (GO:0050660)
Cm32	*COCNU_12G008590*	Ribosomal RNA large subunit methyltransferase E (IPR015507) tRNA (cytidine(32)/guanosine(34)-2-O)-methyltransferase TRM7 (IPR028590)	RNA methylation (GO:0001510) tRNA processing (GO:0008033) methylation (GO:0032259)	methyltransferase activity (GO:0008168) tRNA methyltransferase activity (GO:0008175) glycosyltransferase activity (GO:0016757)
m^5^C34/40/48	*COCNU_13G003290*	RNA (C5-cytosine) methyltransferase (IPR023267) tRNA (C5-cytosine) methyltransferase, NCL1 (IPR023270)	RNA methylation (GO:0001510)	tRNA (cytosine-5-)-methyltransferase activity (GO:0016428) methyltransferase activity (GO:0008168) RNA binding (GO:0003723)
	*COCNU_05G005710*	RNA (C5-cytosine) methyltransferase (IPR023267)	RNA methylation (GO:0001510)	methyltransferase activity (GO:0008168)
	*COCNU_01G013560*	RNA (C5-cytosine) methyltransferase (IPR023267) RNA (C5-cytosine) methyltransferase, putative Rsm-related, plant (IPR023268)	RNA methylation (GO:0001510)	methyltransferase activity (GO:0008168) RNA binding (GO:0003723)
ho^5^U34	*COCNU_16G005830*	tRNA uridine(34) hydroxylase (IPR020936)	No GO Terms	no GO terms
	*COCNU_01G000860*	tRNA uridine(34) hydroxylase (IPR020936)	No GO Terms	no GO terms
ac^4^C34	*COCNU_06G002280*	TmcA/NAT10/Kre33 (IPR032672) RNA cytidine acetyltransferase NAT10 (IPR033688)	rRNA metabolic process (GO:0016072) ncRNA processing (GO:0034470)	acetyltransferase activity (GO:0016407) N-acetyltransferase activity (GO:0008080) ATP binding (GO:0005524)
m^1^G37	*COCNU_06G010450*	tRNA (guanine(37)-N(1))-methyltransferase, eukaryotic (IPR025792)	tRNA methylation (GO:0030488)	tRNA (guanine-N1-)-methyltransferase activity (GO:0009019)
m^1^G9	*COCNU_11G005850*	tRNA (guanine-N1-)-methyltransferase, eukaryotic (IPR007356)	No GO Terms	no GO terms
m^7^G46	*COCNU_15G004650*	tRNA (guanine-N-7) methyltransferase, Trmb type (IPR003358) tRNA (guanine-N-7) methyltransferase catalytic subunit Trm8, eukaryote (IPR025763)	tRNA modification (GO:0006400)	tRNA (guanine-N7-)-methyltransferase activity (GO:0008176)
	*COCNU_15G004660*	tRNA (guanine-N-7) methyltransferase, Trmb type (IPR003358) tRNA (guanine-N-7) methyltransferase catalytic subunit Trm8, eukaryote (IPR025763)	tRNA modification (GO:0006400)	tRNA (guanine-N7-)-methyltransferase activity (GO:0008176)
	*COCNU_07G015760*	tRNA (guanine-N-7) methyltransferase, Trmb type (IPR003358)	tRNA modification (GO:0006400)	tRNA (guanine-N7-)-methyltransferase activity (GO:0008176)
	*COCNU_15G004660*	tRNA (guanine-N-7) methyltransferase, Trmb type (IPR003358) tRNA (guanine-N-7) methyltransferase catalytic subunit Trm8, eukaryote (IPR025763)	tRNA modification (GO:0006400)	tRNA (guanine-N7-)-methyltransferase activity (GO:0008176)
	*COCNU_03G001100*	tRNA (guanine-N(7)-)-methyltransferase non-catalytic subunit (IPR028884)	RNA (guanine-N7)-methylation (GO:0036265)	protein binding (GO:0005515)
m^2^G10	*COCNU_15G003580*	tRNA guanosine-2’-O-methyltransferase, TRM11 (IPR016691)	methylation (GO:0032259)	methyltransferase activity (GO:0008168) nucleic acid binding (GO:0003676)
m^2,2^G26	*COCNU_06G015830*	tRNA methyltransferase, Trm1 (IPR002905)	tRNA processing (GO:0008033)	RNA binding (GO:0003723) tRNA (guanine-N2-)-methyltransferase activity (GO:0004809)
	*COCNU_16G005730*	tRNA methyltransferase, Trm1 (IPR002905)	tRNA processing (GO:0008033)	RNA binding (GO:0003723) tRNA (guanine-N2-)-methyltransferase activity (GO:0004809)
	*COCNU_13G005450*	tRNA methyltransferase, Trm1 (IPR002905)	tRNA processing (GO:0008033)	RNA binding (GO:0003723) tRNA (guanine-N2-)-methyltransferase activity (GO:0004809)
OHyW37	*COCNU_11G006960*	None predicted	No GO Terms	no GO terms
	*COCNU_14G008320*	None predicted	No GO Terms	no GO terms
imG-14(37)	*COCNU_14G010760*	None predicted	No GO Terms	no GO terms
yW/yW-86/yW-72(37)	*COCNU_06G011880*	None predicted	No GO Terms	protein binding (GO:0005515)
Ar(p)64	*COCNU_03G014780*	tRNA A64-2’-O-ribosylphosphate transferase (IPR007306)	Charged-tRNA amino acid modification (GO:0019988)	tRNA A64-2’-O-ribosylphosphate transferase activity (GO:0043399)
s^2^U32	*COCNU_05G004150*	None predicted	No GO Terms	sulfurtransferase activity (GO:0016783)
	*COCNU_14G010500*	Cytoplasmic tRNA 2-thiolation protein 2 (IPR019407)	tRNA thio-modification (GO:0034227) tRNA wobble uridine modification (GO:0002098)	tRNA binding (GO:0000049)
	*COCNU_05G004090*	None predicted	No GO Terms	no GO terms

**Table 3:** Unique fragments for positioning pseudouridines in specific tRNAs.

Position	Found in tRNA	Detected Unique Fragment	Co-Positioning	MS1	MS2
1	Asn9-GUU, Asn13-GUU, Asn17-GUU, Asn18-GUU	p[Ψ-CE]CCUC[I-CE]Gp	I6	*m*/*z* = 1202.137, z = −2	yes
3	Gln2-CUG, Gln3-CUG, Gln5-UUG, Gln6-UUG	[Ψ-CE]UCCAUGp		*m*/*z* = 1135.641, z = −2	yes
3	Gln1-CUG	[Ψ-CE]CCCAU[m^1^G]Gp	m^1^G9	*m*/*z* = 1135.641, z = −2	yes
4	Gln2-CUG, Gln3-CUG, Gln5-UUG, Gln6-UUG	U[Ψ-CE]CCAUGp		*m*/*z* = 1135.641, z = −2	yes
4	Thr1-CGU, Thr2-CGU	CU[Ψ-CE]CCGp		*m*/*z* = 970.620, z = −2	yes
8	Gln1-CUG	UCCCA[Ψ-CE][m^1^G]Gp	m^1^G9	*m*/*z* = 1315.179, z = −2	yes
11	Arg1-UCU, Gly1-UCC, Gly2-UCC, Gly3-UCC, Gly4-UCC, Gly5-UCC	[Ψ-CE]CC[m^1^A]ACGp	m^1^A14	*m*/*z* = 1153.669, z = −2	yes
11	Glu6-UUC	[m^2,2^G][Ψ-CE]CA[m^1^A]Gp	m^2,2^G10, m^1^A14	*m*/*z* = 1035.163, z = −2	yes
12	Met9-CAU	AC[Ψ-CE]C[I-CE]Gp	I14	*m*/*z* = 1021.147, z = −2	yes
25	Gly1-CCC	UAUCA[Ψ-CE]AGp		*m*/*z* = 1312.172, z = −2	yes
25	His2-GUG, His3-GUG, His5-GUG	AA[Ψ-CE]UCCACGp		*m*/*z* = 976.132, z = −3	yes
26	His2-GUG, His3-GUG, His5-GUG	AAU[Ψ-CE]CCACGp		*m*/*z* = 976.132, z = −3	yes
26	Asp10-GUC	UAU[Ψ-CE]UCCGp		*m*/*z* = 1288.660, z = −2	yes
27	Gln3-CUG, Glu2-UUC, Glu4-UUC	ACA[Ψ-CE]CGp		*m*/*z* = 994.141, z = −2	yes
27	Arg1-CCU, Arg2-CCU, Arg3-CCU, Arg4-CCU, Arg2-UCG, Arg2-UCU, Arg3-UCU, Arg4-UCU, Arg5-UCU, Trp1-CCA	C[m^2,2^G][Ψ-CE]CUGp	m^2,2^G26	*m*/*z* = 1004.640, z = −2	yes
28	Gln1-CUG, Gln2-CUG, Gln6-UUG	ACAU[Ψ-CE]Gp		*m*/*z* = 994.634, z = −2	yes
28	Glu1-CUC, Glu2-CUC, Glu3-CUC, Glu4-CUC	AUAC[Ψ-CE]CGp		*m*/*z* = 1147.153, z = −2	yes
29	Gly1-GCC	[Ψ-CE]ACCCUGp		*m*/*z* = 1135.146, z = −2	yes
31	Arg2-ACG, Arg4-ACG, Arg8-ACG	[Ψ-CE][Cm]U[I-CE]CGp	Cm32, I34	*m*/*z* = 990.129, z = −2	yes
31	Ile1-UAU, Ile2-UAU, Ile3-UAU	[Ψ-CE]CU[cmnm^5^Um]AUGp	cmnm^5^Um34	*m*/*z* = 1186.615, z = −2	yes
31	Met5-CAU	[Ψ-CE]CUCAU[m^6^A]Gp	m^6^A37	*m*/*z* = 1307.175, z = −2	yes
32	Ala1-UGC, Ala2-UGC, Ala3-UGC, Ala4-UGC, Pro1-UGG, Pro3-UGG, Pro4-UGG	C[Ψ-CE]U[ncm5U]Gp	ncm^5^U34	*m*/*z* = 847.104, z = −2	yes
33	Arg1-CCG, Arg2-CCG, Arg4-CCG, Arg5-CCG	C[Cm][Ψ-CE]CCGp		*m*/*z* = 977.138, z = −2	yes
33	Gln1-UUG, Gln2-UUG, Gln5-UUG, Gln6-UUG, Gln7-UUG	AC[Ψ-CE]UUGp		*m*/*z* = 983.120, z = −2	yes
35	Asp1-GUC, Asp2-GUC, Asp3-GUC, Asp4-GUC, Asp5-GUC, Asp6-GUC, Asp7-GUC, Asp9-GUC, Asp10-GUC, Asp11-GUC	[Ψ-CE]CA[m^5^C]Gp	m^5^C38	*m*/*z* = 836.623, z = −2	yes
35	Asp8-GUC	[Ψ-CE]CAU[m^1^G]CGp	m^1^G39	*m*/*z* = 1001.646, z = −2	yes
39	Gln1-UUG, Gln2-UUG	[m^6^A]A[Ψ-CE]CUGp	m^6^A37	*m*/*z* = 1001.646, z = −2	yes
39	Cys1-GCA	A[Ψ-CE]CCUUAGp		*m*/*z* = 1300.669, z = −2	yes
40	Ser1-GCU, Ser2-GCU, Ser3-GCU	[Ψ-CE]ACAU[m^7^G]Gp	m^7^G45	*m*/*z* = 1174.168, z = −2	yes
41	Val2-AAC	U[Cm]U[I-CE]ACACAC[Ψ-CE]Gp	Cm32, I34	*m*/*z* = 1312.181, z = −2	yes
47	Lys10-UUU	[m^7^G][Ψ-CE]CAUGp	m^7^G46	*m*/*z* = 1009.639, z = −2	yes
48	Arg5-UCU	AC[Ψ-CE][m^7^G]UGp	m^7^G49	*m*/*z* = 1009.632, z = −2	yes
48	Arg3-UCU	AC[m^7^G]AU[Ψ-CE]Gp	m^7^G45	*m*/*z* = 1174.168, z = −2	yes
49	His2-GUG, His4-GUG, His5-GUG	A[m^7^G]ACC[Ψ-CE]Gp	m^7^G45	*m*/*z* = 1174.168, z = −2	yes
50	Leu3-UAG, Leu4-UAG,	C[I-CE]UC[Ψ-CE]CGp	I47	*m*/*z* = 1162.152, z = −2	yes
55	Gln1-CUG, Gln2-CUG, Gln3-CUG	[m^5^U][Ψ-CE]CA[m^1^A]AUCUCGp	m^5^U54, m^1^A58	*m*/*z* = 1189.163, z = −3	yes
59	Ala-AGC	[m^1^A][Ψ-CE]ACCCCGp	m^1^A58	*m*/*z* = 1306.189, z = −2	yes
59	Ala1-UGC, Ala2-UGC, Ala3-UGC, Ala4-UGC	[m^1^A][Ψ-CE]CCCCUGp	m^1^A58	*m*/*z* = 1294.677, z = −2	yes
60	Arg1-UCG, Arg2-UCG	[m^1^A]C[Ψ-CE]CCCACUGp	m^1^A58	*m*/*z* = 1612.223, z = −2	yes
67	Arg1-CCU, Arg3-CCU	[m^1^A]CCCCUACC[Ψ-CE]Gp	m^1^A58	*m*/*z* = 1176.164, z = −2	yes
67	Arg4-CCU	[m^1^A]CCCUUACC[Ψ-CE]Gp	m^1^A58	*m*/*z* = 1176.164, z = −2	yes
71	Pro2-UGG, Pro5-UGG, Pro6-UGG, Pro7-UGG	UCACCU[Ψ-CE]Gp		*m*/*z* = 1288.164, z = −2	yes
73	Cys2-GCA, Cys4-GCA, Cys5-GCA, Cys6-GCA, Cys8-GCA, Cys9-GCA	CC[m^5^C][Ψ-CE]CCAOH	m^5^C72	*m*/*z* = 1081.677, z = −2	yes

**Table 4:** Unique fragments for positioning inosines in specific tRNAs.

Position	Found in tRNA	Detected Unique Fragment	Co-Positioning	MS1	MS2
5	Gly2-CCC	C[I-CE]ACU[m^1^G]p	m^1^G9	*m*/*z* = 1174.168, z = −2	yes
6	Gly2-CCC	CA[I-CE]CUGp		*m*/*z* = 994.633, z = −2	yes
6	Asn9-GUU, Asn13-GUU, Asn17-GUU, Asn18-GUU	p[Ψ-CE]CCUC[I-CE]Gp	Ψ1	*m*/*z* = 1202.137, z = −2	yes
7	Gln1-CUG	UCCC[I-CE]UGp		*m*/*z* = 1135.641, z = −2	yes
14	Met9-CAU	AC[Ψ-CE]C[I-CE]Gp	Ψ12	*m*/*z* = 1021.147, z = −2	yes
24	Gln2-UUG	C[I-CE]CUUCAGp		*m*/*z* = 1300.669, z = −2	yes
24	Gln7-UUG	C[I-CE]CUC[Ψ-CE]Gp	Ψ28	*m*/*z* = 1162.152, z = −2	yes
28	Met2-CAU, Met3-CAU, Met4-CAU	C[m^2,2^G]U[I-CE]Gp	m^2,2^G26	*m*/*z* = 864.120, z = −2	yes
28	Lys1-UUU, Lys2-UUU, Lys3-UUU, Lys6-UUU, Lys7-UUU, Lys9-UUU	C[m^2,2^G]C[I-CE]UGp	m^2,2^G26	*m*/*z* = 1016.643, z = −2	yes
34	Arg2-ACG, Arg4-ACG, Arg8-ACG, Arg9-ACG	[Ψ-CE][Cm]U[I-CE]CGp	Ψ31, Cm32	*m*/*z* = 1016.641, z = −2	yes
34	Ile1-AAU, Ile2-AAU, Ile3-AAU	CU[I-CE]AUAACGp		*m*/*z* = 1477.198, z = −2	yes
34	Val2-AAC	U[Cm]U[I-CE]ACACAC[Ψ-CE]Gp	Cm32,Ψ41	*m*/*z* = 1312.181, z = −2	yes
37	Glu1-UUC	CU[cmnm^5^Um]UC[I-CE]CCCAGp	cmnm^5^Um34	*m*/*z* = 1205.823, z = −2	yes
46	Asp12-GUC	C[m^7^G][I-CE]UCCGp	m^7^G45	*m*/*z* = 1162.154, z = −2	yes
46	Glu1-CUC, Glu2-CUC, Glu3-CUC	A[m^7^G][I-CE]CCCGp	m^7^G45	*m*/*z* = 1174.168, z = −2	yes
47	Leu3-UAG, Leu4-UAG	C[I-CE]UC[Ψ-CE]CGp	Ψ50	*m*/*z* = 1162.152, z = −2	yes
48	Glu4-CUC	A[m^7^G]A[I-CE]CUGp	m^7^G46	*m*/*z* = 1186.166, z = −2	yes
59	Ser1-UGA	[m^1^A][I-CE]CCCCGp	m^1^A58	*m*/*z* = 1153.665, z = −2	yes
65	Arg1-UCU	[I-CE]CAAACGp		*m*/*z* = 1170.640, z = −2	yes
67	Gly2-GCC, Gly3-GCC	CU[I-CE]UCCGp		*m*/*z* = 1135.639, z = −2	yes

**Table 5:** The size of each chromosome of *C. nucifera* L. (2n = 32).

Chromosome Number	Length/bp
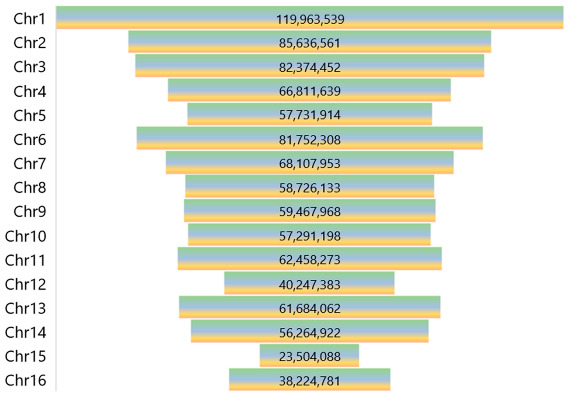	

## Data Availability

The data that support the findings of this study are available from the corresponding author upon reasonable request.

## Supplementary Materials

The following supporting information can be downloaded at: https://www.mdpi.com/article/10.3390/genes14061287/s1, List 1: Cocos_nucifera_cytoplasm_tRNAs; List 2: Cocos_nucifera_chloroplast_tRNAs; List 3: Cocos_nucifera_mitochondrion_tRNAs. All tRNA sequences were mined by tRNAscan-SE 2.0 searching and manual confirmation.
